# Genome Sequence of a *Klebsiella pneumoniae* Sequence Type 258 Isolate with Prophage-Encoded *K. pneumoniae* Carbapenemase

**DOI:** 10.1128/genomeA.00659-15

**Published:** 2015-06-18

**Authors:** Liang Chen, Kalyan D. Chavda, Frank R. DeLeo, Kendall A. Bryant, Michael R. Jacobs, Robert A. Bonomo, Barry N. Kreiswirth

**Affiliations:** a Public Health Research Institute, New Jersey Medical School, Rutgers University, Newark, New Jersey, USA; b Laboratory of Human Bacterial Pathogenesis, Rocky Mountain Laboratories, National Institute of Allergy and Infectious Diseases, National Institutes of Health, Hamilton, Montana, USA; c Orlando Health, Orlando, Florida, USA; d Department of Pathology, Case Western Reserve University and University Hospitals Case Medical Center, Cleveland, Ohio, USA; e Louis Stokes Cleveland Department of Veterans Affairs Medical Center, Research Service, Cleveland, Ohio, USA; f Departments of Medicine, Pharmacology, Molecular Biology and Microbiology, Case Western Reserve University, Cleveland, Ohio, USA

## Abstract

We present the draft genome sequence of a *Klebsiella pneumoniae* carbapenemase (KPC)-producing sequence type 258 (ST258) *K. pneumoniae* strain, ST258_FL. Uniquely, strain ST258_FL harbors two copies of the *bla*_KPC_ gene on the chromosome, one of which is integrated into a prophage.

## GENOME ANNOUNCEMENT

*Klebsiella pneumoniae* carbapenemase (KPC)-producing bacteria have spread worldwide and pose a significant threat in hospital settings (1). The gene encoding KPC, *bla*_KPC_, is harbored by a Tn*3*-like transposon, Tn*4401*, and is carried by various transferable plasmids (1). Unlike other carbapenemase genes, such as *bla*_NDM_, the chromosomal integration of *bla*_KPC_ is rare (2, 3).

The *K. pneumoniae* strain ST258_FL was isolated from the urine of a patient in a medical center in Orlando, Florida, in 2008. The strain belongs to multilocus sequence type 258 (ST258), an epidemic KPC-producing clone in the United States and worldwide (1). Strain ST258_FL was resistant to multiple antibiotics used clinically, including β-lactams, fluoroquinolones, aminoglycosides, and trimethoprim-sulfamethoxazole but was susceptible to colistin, polymyxin B, and tigecycline.

Next-generation sequencing was performed using an Illumina MiSeq instrument with 150-bp paired-end reads and ~680-fold coverage. *De novo* assembly was accomplished by using a combination of A5-miseq (4), Velvet (5), and CLC genomic workbench 8.0. These contigs were then ordered by genome position and orientation using previous published NJST258_1 and NJST258_2 genomes (6) as references in Geneious 7.17 to create supercontigs, resulting in 12 supercontigs ranging from 2,669 to 5,391,203 bp. These supercontigs were annotated using the Prokaryotic Genomes Automatic Annotation Pipeline (PGAAP) (http://www.ncbi.nlm.nih.gov/genome/annotation_prok/), providing a total of 5,914 genes, 5,748 coding DNA sequence genes, 25 rRNAs (5S, 16S, and 23S), 87 tRNAs, and 5 noncoding RNAs (ncRNAs).

*In silico* genome analysis identified 16 antimicrobial resistance genes encoding resistance to β-lactams (*bla*_KPC-3_, *bla*_SHV-11_, *bla*_OXA-9_, and *bla*_TEM-1_), aminoglycosides [*aph(4)-Ia*, *aac(3)-IVa*, *aac(6)-Ib,* and *aph(3′)-Ia*], fluoroquinolones (*oqxA* and *oqxB*), macrolides (*mphA*), phenicols (*catA1* and *cmlA1*), and sulfonamide-trimethoprim (*sul1, sul3*, and *dfrA12*). Further analysis discovered mutations encoding amino acid substitutions at Ser83-Ile and Asp87-Asn within the quinolone resistance-determining (QRDR) regions of GyrA, and Ser80-Ile of ParC. Examination of the outer membrane protein OmpK35 and OmpK36 genes revealed an extra serine insertion at amino acid 213 in OmpK36, and a premature stop codon at amino acid 89 in OmpK35, respectively. In addition, five plasmid groups were identified in strain ST258_FL, belonging to incompatibility groups ColE1, FII_K_, R, I2, and FIB_K_.

Two copies of *bla*_KPC_ harboring Tn*4401b* were identified in the genome of strain ST258_FL. Interestingly, both copies were located on the chromosome and not on a plasmid. Manual inspection of the *de novo* assembly showed paired-end reads spanning the junctions between the Tn*4401b* and the chromosome. In addition, the chromosomal integrated *bla*_KPC_ genes were confirmed using PCR targeted to the junctions between Tn*4401b* and the chromosome for both copies. One copy of *bla*_KPC_-harboring Tn*4401b* is integrated into a DNA-binding protein gene (KPNJ1_03166 at genome of NJST258_1) in prophage 258.4. The second copy of Tn*4401b* is inserted as an inversion 23,762 bp downstream of the first Tn*4401b*.

In conclusion, we present the sequence of a KPC-producing ST258 *K. pneumoniae* isolate, with two copies of chromosomally integrated *bla*_KPC_ genes. One of the *bla*_KPC_ genes is contained in a prophage, raising the possibility that *bla*_KPC_ may be transferred through transduction if the prophage is activated. This unique insertion has not been previously reported.

### Nucleotide sequence accession number.

The draft genome sequence of *Klebsiella pneumoniae* strain ST258_FL has been included in the GenBank whole-genome shotgun (WGS) database under the accession no. LAKK00000000.
